# Supplementary material for: Transcriptomics, regulatory syntax, and enhancer identification in mesoderm-induced ESCs at single-cell resolution
Source: Cell Rep. Author manuscript; Available in PMC 2022 Nov 9. (PMC9644345; doi:10.1016/j.celrep.2022.111219)
Supplement: 1 [file NIHMS1831963-supplement-1.pdf]

Cell Reports, Volume *40*

Supplemental information

Transcriptomics, regulatory syntax, and enhancer identification in mesoderm-induced ESCs at single-cell resolution

Mamduh Khateb, Jelena Perovanovic, Kyung Dae Ko, Kan Jiang, Xuesong Feng, Natalia Acevedo-Luna, Jérome Chal, Veronica Ciuffoli, Pavol Genzor, James Simone, Astrid D. Haase, Olivier Pourquié, Stefania Dell'Orso, and Vittorio Sartorelli

Figure S1

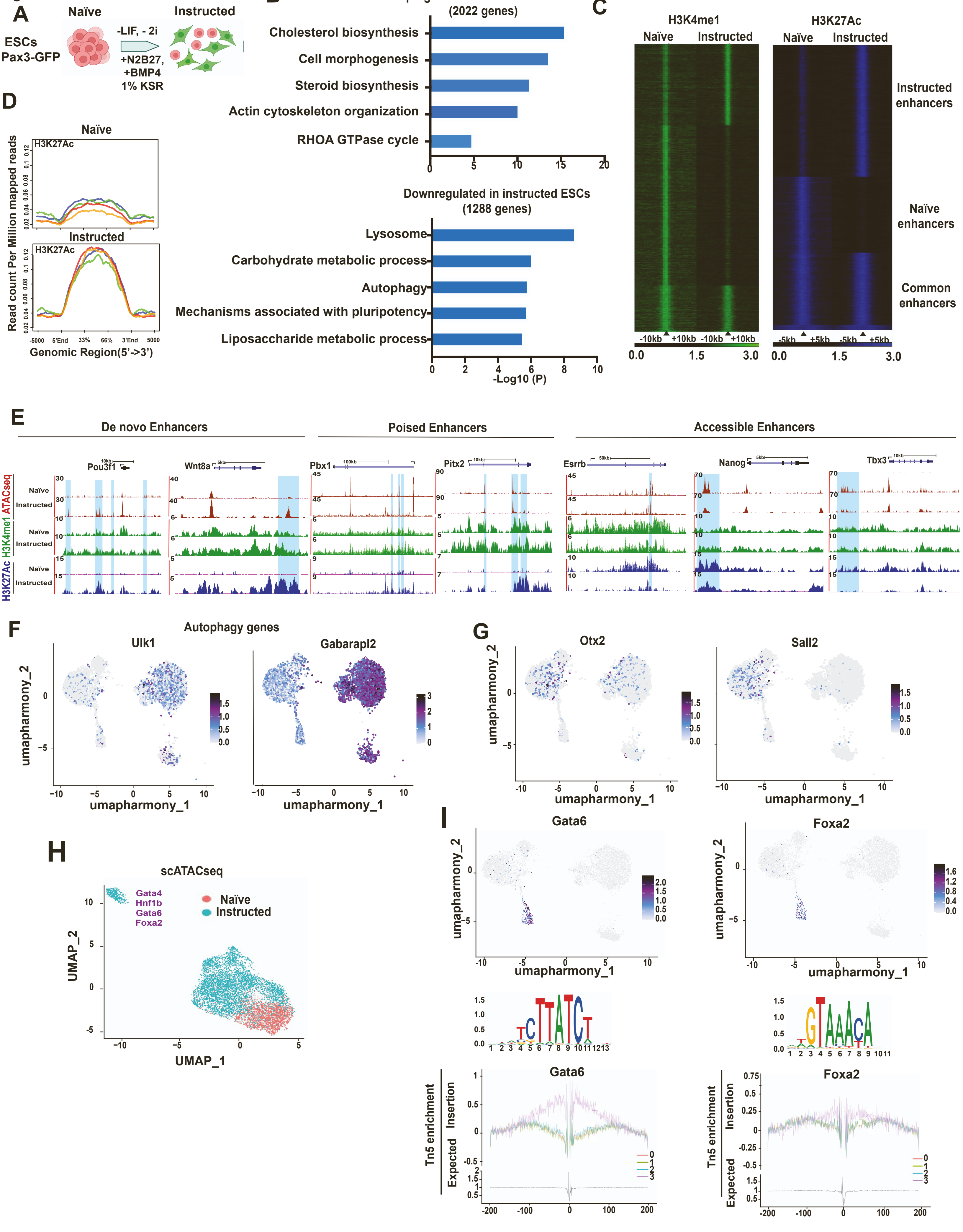

A
Naïve
Instructed
ESCs
Pax3-GFP
-LIF, - 2i
+N2B27,
+BMP4
1% KSR
B
Upregulated in instructed ESCs
(2022 genes)
Cholesterol biosynthesis
Cell morphogenesis
Steroid biosynthesis
Actin cytoskeleton organization
RHOA GTPase cycle
Downregulated in instructed ESCs
(1288 genes)
Lysosome
Carbohydrate metabolic process
Autophagy
Mechanisms associated with pluripotency
Liposaccharide metabolic process
-Log10 (P)
C
H3K4me1
H3K27Ac
Naïve
Instructed
Instructed enhancers
Naïve enhancers
Common enhancers
-10kb
+10kb
-5kb
+5kb
D
Naïve
Instructed
H3K27Ac
Read count Per Million mapped reads
Genomic Region(5'->3')
E
De novo Enhancers
Poised Enhancers
Accessible Enhancers
Pou3f1
Wnt8a
Pbx1
Pitx2
Esrrb
Nanog
Tbx3
ATACseq
H3K4me1
H3K27Ac
F
Autophagy genes
Ulk1
Gabarapl2
umapharmony_2
umapharmony_1
G
Otx2
Sall2
H
scATACseq
Gata4
Hnf1b
Gata6
Foxa2
Naïve
Instructed
UMAP_2
UMAP_1
I
Gata6
Foxa2
Tn5 enrichment
Insertion
Expected

Supplemental Figure S1 (related to figure 1): Enhancers Modalities and Single Cell Analysis of Transcriptome and Chromatin Accessibility in Naïve and Instructed ESCs.

(A) Scheme of ESCs Pax3-GFP cultured in N2B27 medium supplemented with Bmp4 and 1% KSR without LIF and 2i for 48 hr (Instructed). (B) Selected gene ontology terms of upregulated (upper panel) and downregulated (lower panel) transcripts in instructed ESCs. (C) H3K4me1 and H3K27ac heatmaps in naïve and instructed ESCs. Interval is -10Kb/+10Kb and -5Kb/+5Kb from the center of the peak signal, respectively. (D) Averaged normalized tag intensities of H3K27ac at naïve and instructed regulatory regions. (E) Genome browser representation of ATAC-seq and ChIP-seq tracks at Pou3f1, Wnt8, Pbx1, Pitx2, Esrrb, Nanog, and Tbx3 loci. (F) Expression of autophagy Ulk1 and Gabarapl2 transcripts in scRNA-seq naïve and instructed ESCs clusters. (G) Expression of Otx2 and Sall2. (H) scATAC-seq of naïve and instructed ESCs clusters. Gata4, Gata6, Hnf1b, and Foxa2 TSS were accessible and footprinted in the indicated instructed cluster. (I) Expression of Gata6 and Foxa2 transcripts (top panel), motif enrichment and overall scATAC-seq Gata6 and Foxa2 footprinting. The x-axis in the footprinting represents nucleotides from the DNA binding motif located at “0”.

Figure S2

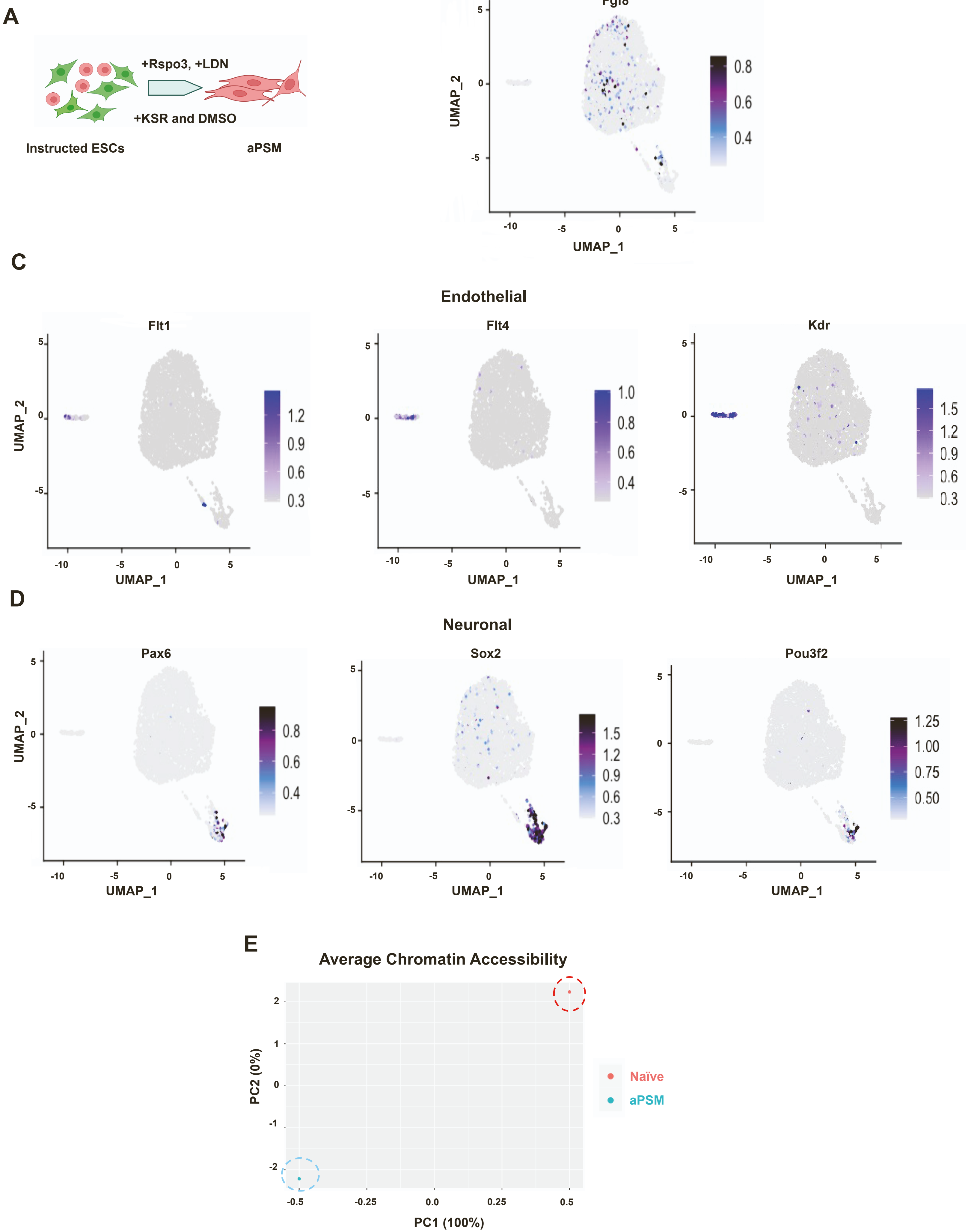
A
+Rspo3, +LDN
+KSR and DMSO
Instructed ESCs
aPSM
B
Fgf8
UMAP_2
UMAP_1
0.8
0.6
0.4
C
Endothelial
Flt1
Flt4
Kdr
UMAP_2
UMAP_1
D
Neuronal
Pax6
Sox2
Pou3f2
UMAP_2
UMAP_1
E
Average Chromatin Accessibility
PC2 (0%)
PC1 (100%)
Naïve
aPSM

Supplemental Figure S2 (related to figure 2): Single Cell Gene Expression of Endothelial and Neuronal Factors in Pax3-GFP-Positive ESCs.

(A) Scheme indicating differentiating medium conditions to instruct ESCs to acquire an anterior presomitic mesoderm (aPSM) fate. (B) Fgf8 expression in aPSM cells. (C) Expression of endothelial transcripts Flt1, Flt4, and Kdr in aPSM cells. (D) Expression of neuronal transcripts Pax6, Sox2, and Pou3f2 in aPSM cells. (E) PCA plot of average chromatin accessibility in naïve ESCs and aPSM cells.

Figure S3

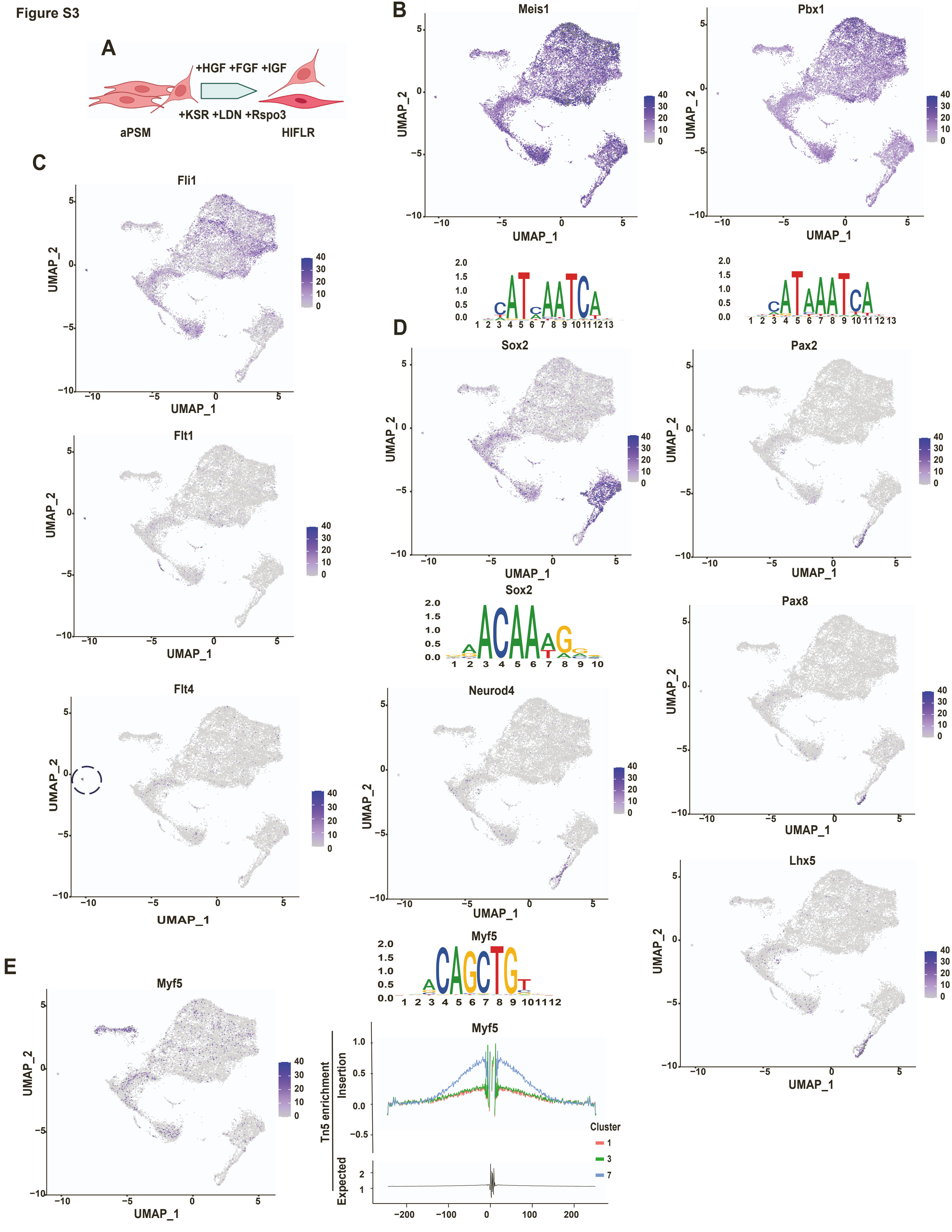

A
+HGF +FGF +IGF
+KSR +LDN +Rspo3
aPSM
HIFLR
B
Meis1
Pbx1
UMAP_1
UMAP_2
C
Fli1
Flt1
Flt4
D
Sox2
Pax2
Pax8
Neurod4
Lhx5
Myf5
E
Tn5 enrichment
Insertion
Expected
Cluster
1
3
7

Supplemental Figure S3 (related to figure 3): Single Cell Omics of Neuronal and Myogenic Markers.

(A) Scheme indicating medium conditions to induce aPSM cells to acquire an initial myogenic cell fate. (B) Expression and DNA binding motifs for Meis1 and Pbx1. (C) Fli1, Flt1, and Flt4 expression. (D) Expression and DNA binding motif for Sox2, and expression of Pax2, Neurod4, Pax8, and Lhx5. (E) Expression (left panel), DNA binding motif, and footprinting (right panel) for Myf5. The x-axis in the footprinting represents nucleotides from the DNA binding motif located at “0”.

Figure S4

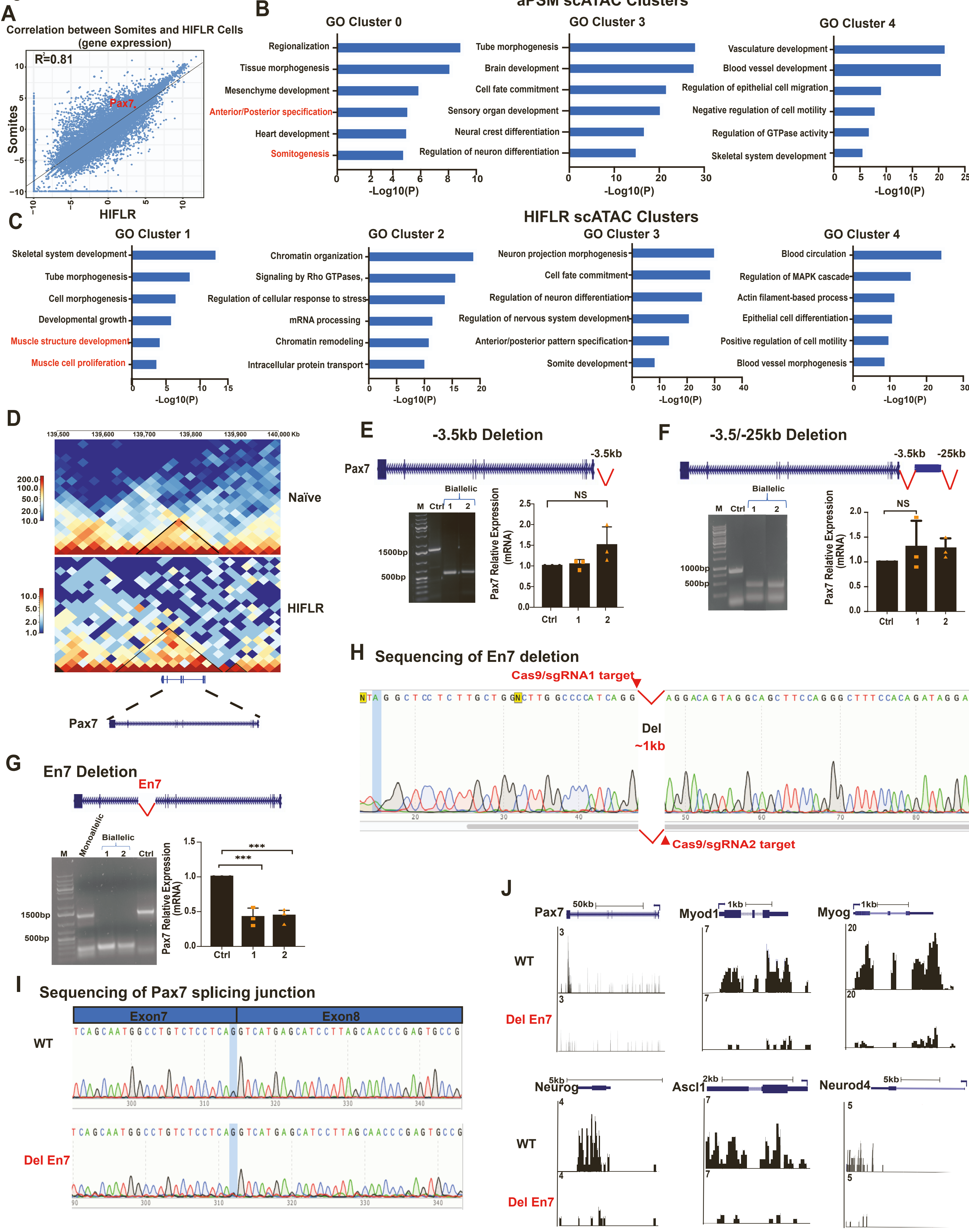
A
Correlation between Somites and HIFLR Cells
(gene expression)
R²=0.81
Pax7
Somites
HIFLR
B
aPSM scATAC Clusters
GO Cluster 0
Regionalization
Tissue morphogenesis
Mesenchyme development
Anterior/Posterior specification
Heart development
Somitogenesis
-Log10(P)
GO Cluster 3
Tube morphogenesis
Brain development
Cell fate commitment
Sensory organ development
Neural crest differentiation
Regulation of neuron differentiation
-Log10(P)
GO Cluster 4
Vasculature development
Blood vessel development
Regulation of epithelial cell migration
Negative regulation of cell motility
Regulation of GTPase activity
Skeletal system development
-Log10(P)
C
HIFLR scATAC Clusters
GO Cluster 1
Skeletal system development
Tube morphogenesis
Cell morphogenesis
Developmental growth
Muscle structure development
Muscle cell proliferation
-Log10(P)
GO Cluster 2
Chromatin organization
Signaling by Rho GTPases,
Regulation of cellular response to stress
mRNA processing
Chromatin remodeling
Intracellular protein transport
-Log10(P)
GO Cluster 3
Neuron projection morphogenesis
Cell fate commitment
Regulation of neuron differentiation
Regulation of nervous system development
Anterior/posterior pattern specification
Somite development
-Log10(P)
GO Cluster 4
Blood circulation
Regulation of MAPK cascade
Actin filament-based process
Epithelial cell differentiation
Positive regulation of cell motility
Blood vessel morphogenesis
-Log10(P)
D
139,500 139,600 139,700 139,800 139,900 140,000 Kb
Naïve
HIFLR
Pax7
E
-3.5kb Deletion
Pax7
-3.5kb
Biallelic
M Ctrl 1 2
1500bp
500bp
Pax7 Relative Expression (mRNA)
NS
Ctrl 1 2
F
-3.5/-25kb Deletion
-3.5kb -25kb
Biallelic
M Ctrl 1 2
1000bp
500bp
Pax7 Relative Expression (mRNA)
NS
Ctrl 1 2
G
En7 Deletion
En7
M Monoallelic Biallelic 1 2 Ctrl
1500bp
500bp
Pax7 Relative Expression (mRNA)
***
***
Ctrl 1 2
H
Sequencing of En7 deletion
Cas9/sgRNA1 target
Del
~1kb
Cas9/sgRNA2 target
I
Sequencing of Pax7 splicing junction
Exon7
Exon8
WT
Del En7
J
Pax7 50kb
Myod1 1kb
Myog 1kb
WT
Del En7
Neurog 5kb
Ascl1 2kb
Neurod4 5kb
WT
Del En7

Supplemental Figure S4 (related to figure 4): Identification and Characterization of Pax7 En7 Enhancer.

(A) Gene expression correlation plot of somites and HIFLR cells RNA-seq. (B) Selected gene ontology of scATAC-seq clusters for aPSM and (C) for HIFLR cells. (D) Heatmap of contact matrices from Hi-C data indicating that Pax7 locus resides in a topologically associating domain in both naïve ESCs and HIFLR cells. (E) Gel electrophoresis of genomic DNA documenting biallelic deletion of the -3.5Kb Pax7 region. Quantitative PCR was employed to measure Pax7 mRNA expression in control and two independent biallelic deleted ESC clones (n=3, NS, not significant). (F) Gel electrophoresis of genomic DNA documenting biallelic deletion of the -3.5Kb and -25Kb Pax7 regions. Pax7 mRNA expression in control and two independent biallelic deleted -3.5Kb/-25Kb ESC clones (n=3, NS, not significant). (G) Gel electrophoresis of genomic DNA documenting monoallelic (one clone) and biallelic deletion of En7 Pax7 region in two independent clones. Quantitative PCR was employed to measure Pax7 mRNA in control and two independent biallelic E7 deleted ESC clones. Data are represented as mean -/+SD (n=3). Significance is displayed as $p<0.001$ (***). (H) DNA sequencing chromatogram of a En7 deleted ESC clone. (I) Sequencing chromatogram of cDNA derived from RNA of WT (upper) or En7 deleted ESC clone (lower). Pax7 exon 7 and exon 8 sequences are shown. (J) RNA-seq tracks of Pax7, Myod1, Myogenin, Neurogenin, Ascl1, and Neurod4 in control (WT) and En7 deleted ESCs.
